# Changes in Microbiota Profiles After Prolonged Frozen Storage of Stool Suspensions

**DOI:** 10.3389/fcimb.2020.00077

**Published:** 2020-02-28

**Authors:** Stéphane Dorsaz, Yannick Charretier, Myriam Girard, Nadia Gaïa, Stefano Leo, Jacques Schrenzel, Stephan Harbarth, Benedikt Huttner, Vladimir Lazarevic

**Affiliations:** ^1^Genomic Research Laboratory, Division of Infectious Diseases, Department of Medicine, Geneva University Hospitals and Faculty of Medicine, Geneva, Switzerland; ^2^Bacteriology Laboratory, Division of Laboratory Medicine, Department of Diagnostics, Geneva University Hospitals and Faculty of Medicine, Geneva, Switzerland; ^3^Infection Control Program, Division of Infectious Diseases, Geneva University Hospitals and Faculty of Medicine, Geneva, Switzerland

**Keywords:** long-term frozen storage, stool, taxonomic profiling, DNA extraction, microbiota, fecal microbiota transplantation

## Abstract

**Introduction:** Fecal microbiota transplantation (FMT) is recommended as safe and effective treatment for recurrent *Clostridioides difficile* infections. Freezing the FMT preparation simplifies the process, allowing a single stool sample to be used for multiple receivers and over an extended period of time. We aimed to assess the effect of long-term frozen storage on bacterial taxonomic profiles of a stool suspension prepared for FMT.

**Methods:** DNA was extracted from a stool suspension before freezing and sequentially during the 18-month storage period at −80°C. Two different protocols were used for DNA extraction. The first relied on a classical mechanical and chemical cell disruption to extract both intra- and extracellular DNA; the second included specific pre-treatments aimed at removing free DNA and DNA from human and damaged bacterial cells. Taxonomic profiling of bacterial communities was performed by sequencing of V3–V4 16S rRNA gene amplicons.

**Results:** Microbiota profiles obtained by whole DNA extraction procedure remained relatively stable during frozen storage. When DNA extraction procedure included specific pre-treatments, microbiota similarity between fresh and frozen samples progressively decreased with longer frozen storage times; notably, the abundance of Bacteroidetes decreased in a storage duration-dependent manner. The abundance of Firmicutes, the main butyrate producers in the colon, were not much affected by frozen storage for up to 1 year.

**Conclusion:** Our data show that metataxonomic analysis of frozen stool suspensions subjected to specific pre-treatments prior to DNA extractions might provide an interesting indication of bacterial resistance to stress conditions and thus of chances of survival in FMT recipients.

## Introduction

Fecal microbiota transplantation (FMT), consisting in the transfer of feces of a healthy donor to the gastrointestinal tract of a receiver, is recommended both by the European Society for Microbiology and Infectious Diseases, and the American College of Gastroenterology, as an efficacious alternative to standard antibiotic treatment for recurrent *Clostridioides difficile* infection (CDI) (Surawicz et al., 2013; van Nood et al., 2013; Debast et al., 2014). The results of a small trial suggested that FMT could even be an alternative strategy to standard vancomycin regimen in primary CDI (Juul et al., 2018). The potential benefit of FMT in other conditions, including inflammatory bowel disease, metabolic syndrome, and cancer is being investigated in 160 registered clinical trials (NIH, ClinicalTrials.gov, December 11, 2018).

Next-generation sequencing (NGS) techniques based on whole metagenome shotgun sequencing (metagenomics) or on the sequencing of a taxonomic marker such as the 16S rRNA gene (metataxonomics) enable microbiologists to assess the composition of microbial communities at different levels of taxonomic hierarchy. A metataxonomic analysis confirmed the benefit of FMT in recovering “normal” microbiota in recurrent CDI patients with dysbiotic lowered-diversity microbial communities (Song et al., 2013).

In clinical practice, the recruitment, screening, and selection of donors and preparing their fresh fecal material for FMT is labor-intensive and costly. Freezing of donors' stool samples preparation simplifies the FMT process since a single stool sample might be used across multiple receivers and over an extended period. FMT using frozen sample preparations stored up to 6 months has been shown to be clinically as effective as fresh samples for recurrent CDI treatment (Hamilton et al., 2013; Youngster et al., 2014; Costello et al., 2015; Satokari et al., 2015; Cheminet et al., 2018). However, the impact of frozen storage—that may depend on stool processing techniques (Bahl et al., 2012; Burz et al., 2019; Papanicolas et al., 2019)—on the FMT efficacy for conditions other than CDI remains to be determined.

In our study, we aimed to assess the effect of long-term frozen storage (from 1 week up to 18 months) on bacterial taxonomic profiles of stool sample suspension prepared for FMT. Two different methods were used for DNA extraction at each time point analyzed. One of them relied on a classical mechanical and chemical cell disruption to extract whole (extra- and intracellular) DNA (method NS-MAG); the second (MOLZ), included enzymatic (proteinase K), chemical (patented by Molzym), and DNase pre-treatments to remove DNA from human and damaged bacterial cells, prior to extraction of intracellular DNA from intact bacterial cells. We used MOLZ procedure to identify and quantify bacterial populations that were resistant to stress conditions and thus would have better chances of survival in FMT recipients.

## Materials and Methods

### Samples Handling and DNA Extraction

Stool sample(s) collected from a healthy volunteer were prepared based on the protocol described in Youngster et al. (2014) and used in a large randomized controlled trial (Clinicaltrials.gov, NCT02472600) (Huttner et al., 2019). Briefly, fresh stool was suspended in NaCl 0.9% (6 mL per g of stool) using a commercial blender and sequentially sieved using 1, 0.5, and 0.25-mm Nylon filters (NITEX, Milian). The suspension was centrifuged at 2,800 × g (Hettich Rotanta 460RS) for 20 min and the supernatant was discarded. Cold 80% glycerol in NaCl 0.9% was added to fecal concentrate at one tenth of the volume of the initial NaCl stool suspension. The final suspension was fractionated into 50-μL aliquots, which were either immediately subjected to DNA extraction (time 0) or kept frozen at −80°C.

Frozen aliquots of the final stool suspension were thawed after storage for 1 week, 1 month, and then every 3 months from month 3 to month 18. At each time point, two DNA extraction procedures were used, each in triplicate, starting from the whole 50-μL aliquot. The first (MOLZ) was performed using an Ultra-Deep Microbiome Prep kit (Molzym) for selective enrichment of bacterial/fungal DNA according to the manufacturer's instructions for tissue samples (includes a proteinase K pre-treatment). DNA was recovered in 100 μL of dd-H_2_O. The second (NS-MAG) protocol (with no bacterial/fungal DNA enrichment) consisted in: (i) a 20-min bead-beating mechanical disruption in a Nucleospin Bead Tube (Macherey-Nagel), (ii) chemical lysis and DNA purification with a MagCore Genomic DNA Tissue Kit (RBC Bioscience), and (iii) DNA elution with 60 μL of Tris–HCl (10 mM pH = 8), as described previously (Lazarevic et al., 2018). Negative extractions controls were not necessary since the bacterial DNA burden in DNA extracts was > 4 orders of magnitude higher relative to the typical DNA yield of blanks obtained using the same DNA extraction protocols (Lazarevic et al., 2018).

### Quantification of DNA

qPCR quantification of DNA was performed using primers targeting the V3 region of bacterial 16S rRNA genes (*E*. *coli* positions 338–534) on an Mx3005P qPCR system with Brilliant III SYBR Green QPCR Master Mix (Agilent Technologies) as previously described (Lazarevic et al., 2016). One microliter of a 1:1,000 diluted DNA extract was added to the qPCR reaction mix. The cycling conditions included an initial step of 10 min at 95°C followed by 40 cycles of 95°C for 5 s and 68° for 20 s. All reactions were carried out in duplicate and the reference curve for DNA absolute quantitation were obtained using known concentrations of genomic DNA of *E*. *coli* strain DH5α. The number of 16S rRNA gene copies was calculated considering that 1 pg of *E*. *coli* DH5α DNA corresponds to 1,493 copies of the 16S rRNA gene. Fluorometric DNA quantitation was performed using Qubit dsDNA BR Assay kit (Invitrogen). DNA load in NS-MAG extracts was corrected for volume loss during DNA extraction.

### 16S Amplicon Sequencing

Primers 341F 5′-CCTACGGGNGGCWGCAG-3′ and 805R 5′-GACTACHVGGGTATCTAAKCC-3′ (Herlemann et al., 2011), targeting the V3–V4 region of the bacterial 16S rRNA genes (*E*. *coli* positions 341–805) were used to generate amplicons starting with 2 ng of extracted bacterial DNA and using the KAPA2G Robust HotStart ReadyMix (Kapa Biosystems). In a no-template control, DNA extract was replaced by dd-H_2_O. PCR conditions were: initial denaturation step of 3 min at 95°C; 30 cycles of 30 s at 95°C, 30 s at 51°C and 60 s at 72°C; final extension step of 10 min at 72°C. Each sample was PCR amplified in duplicate that were subsequently combined and the DNA quality and quantity was determined on a 2100 Bioanalyzer (Agilent Technologies).

The amplicon barcoding/purification and construction of the sequencing library were performed as described previously (Lazarevic et al., 2016). Sequencing was carried out for 2 × 300 cycles on an Illumina MiSeq instrument using MiSeq v3 Reagent Kit at LGC Genomics (Berlin, Germany). Demultiplexed fastq files were generated from base-calls using Illumina's bcl2fastq v.2.17.1.14 software. Clipping of sequencing adapter remnants and primer sequences was performed using proprietary LGC Genomics software. Sequencing data were submitted to European Nucleotide Archive (ENA) database under study number PRJEB33794.

### Bioinformatics Analysis

Forward and reverse-complemented reads were quality filtered and merged with PEAR v.0.9.11 (Zhang et al., 2014) with the following settings: maximum assembly length (-m) = 460; minimum assembly length (-n) = 390; minimum overlap (-v) = 20; minimum read size after trimming (-t) = 230; *p*-value (-p) = 0.0001; maximal proportion of uncalled bases (-u) = 0; and quality score threshold in trimming (-q) = 33. The merged sequences were then subjected to the UPARSE pipeline (USEARCH v.8.1.64) (Edgar, 2013) with the following steps: reads length filtering and quality filtering (-fastq_filter); dereplication (-derep_fulllength); clustering into operational taxonomic units (OTUs) (-cluster_otus). Taxonomic assignment of the representative OTUs was performed in MOTHUR v.1.35.1 using classify.seqs command (method = wang; cutoff = 80) and the EzBioCloud 16S rRNA gene sequence database (Yoon et al., 2017). From 535 identified OTUs, we removed those that (i) had a relative abundance higher in the no-template control than in the samples (*n* = 13) or (ii) were assigned to eukaryotes or remained taxonomically unassigned (*n* = 141). A final OTU table with 381 taxonomic entries was obtained. Normalization of OTUs counts for sequencing depth and taxonomic binning analysis were performed using the RHEA pipeline in R.

### Absolute Abundance of OTUs

The number of 16S rRNA gene copies (absolute abundance) of OTUs was calculated by multiplying their respective relative abundance (obtained from 16S NGS data) by the total number of 16S rRNA gene copies (determined by qPCR) in the same sample.

### Clustering of Bacterial Communities

Bacterial community comparisons were based on Bray-Curtis similarity (Bray and Curtis, 1957), which is a non-phylogenetic metric. Principal coordinates analysis (PCoA) of Bray-Curtis similarities, was performed in PRIMER (Primer-E Ltd.).

## Results

**Figure 1:**
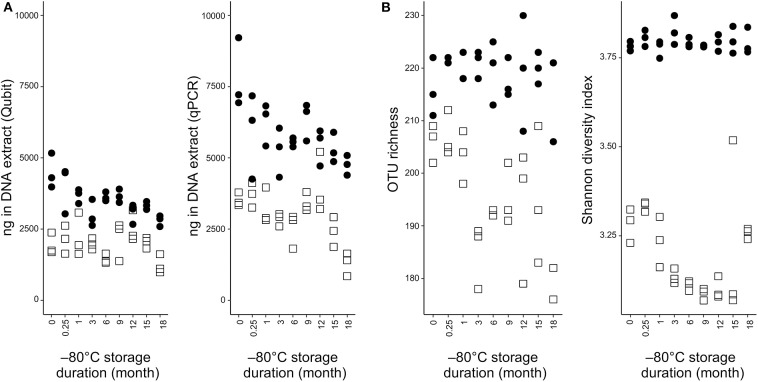
DNA quantification and alpha diversity indexes in NS-MAG and MOLZ extracts. **(A)** DNA load determined by fluorometric quantitation or qPCR targeting bacteria. **(B)** Effect of −80°C storage duration on alpha diversity indices. Filled circles, NS-MAG-treated samples; empty squares, MOLZ-treated samples.

DNA concentrations related to frozen storage period of the samples processed by the two extraction methods were assessed by both qPCR and DNA-binding fluorescent dyes quantification of DNA (Figure 1A). The most noticeable difference was the extracted DNA quantity, which was about twice more abundant in the NS-MAG samples at all observation times, regardless the DNA quantification method. DNA extracts obtained after 18-month cryopreservation had the lowest mean DNA concentration for both extraction methods.

Sequencing of 16S rRNA gene V3–4 rRNA amplicon libraries generated 20,247,102 raw reads. Following quality filtering, the number of merged pairs of reads was normalized to 13,274, as this was the lowest read count found for any sample aliquot. Using the UPARSE pipeline, 381 unique OTUs were identified.

**Figure 2:**
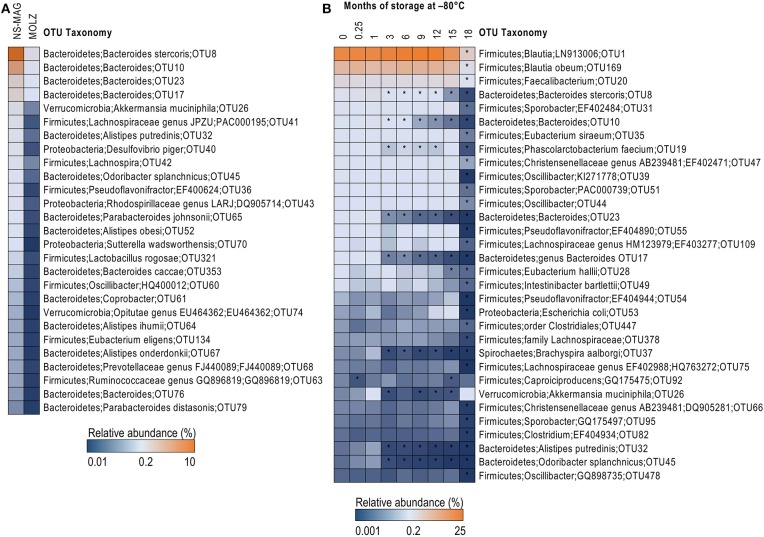
Taxonomic differences between NS-MAG and MOLZ extracts observed at baseline and following frozen storage. **(A)** Difference in relative abundance of OTUs between fresh NS-MAG and fresh MOLZ samples. Only OTUs with average relative abundance >0.1% in NS-MAG samples and a >5-fold reduction in the relative abundance in MOLZ- compared to NS-MAG-treated samples are presented. The mean values of three replicates are shown. **(B)** Changes in the relative abundance of OTUs during frozen storage observed using the MOLZ DNA extraction protocol. Only OTUs with average relative abundance >0.05% at baseline (fresh sample, 0 month) and a >50% reduction in the relative abundance (asterisk) at any of the time points during frozen storage compared to baseline are presented. The mean values of three replicates extracted on indicated time point are given.

The effect of DNA extraction method on fecal microbiota alpha diversity was assessed by calculating OTU richness (number of OTUs identified) and Shannon diversity index. Both alpha diversity indices were higher in NS-MAG than in MOLZ extracted samples (Figure 1B) and they showed less variation in NS-MAG samples. All of the 81 OTUs with an average relative abundance >0.1% in fresh NS-MAG samples were identified in fresh MOLZ-treated samples but with up to 16-fold decreased (Figure 2A) or up to 4-fold increased relative abundance. Most of those depleted OTUs were from the phylum Bacteroidetes.

**Figure 3:**
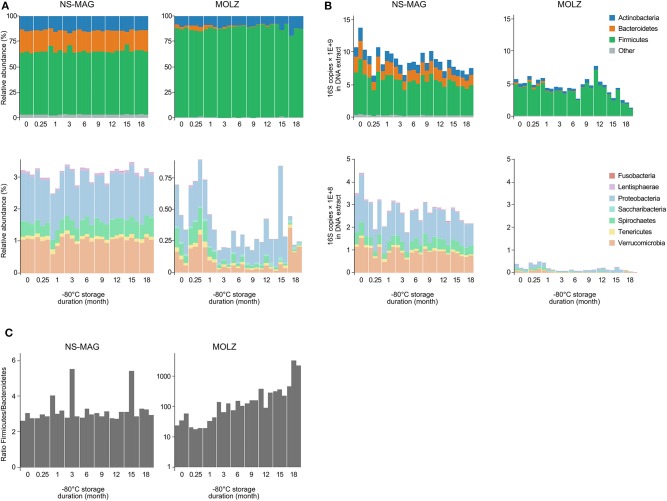
Relative and absolute abundance of bacterial taxa. **(A)** Relative abundance of 16S amplicons assigned to bacterial phyla. **(B)** Absolute abundance of 16S amplicons expressed as number of 16S rRNA gene copies assigned to bacterial phyla. **(C)** Firmicutes to Bacteroidetes 16S rRNA gene copy ratio.

In agreement with known human fecal microbiota composition, the most abundant bacterial phyla identified by taxonomic assignment were Firmicutes, Actinobacteria, Bacteroidetes, Proteobacteria, Verrucomicrobia, Spirochaetes, and Tenericutes ordered by decreasing relative abundance in NS-MAG samples (four panels of Figure 3A). Comparison of MOLZ to NS-MAG samples revealed higher proportions of Firmicutes and lower relative abundances of other phyla at all time points. For example, the relative abundance of Bacteroidetes was on average 21.7% in fresh NS-MAG but only 2.5% in fresh MOLZ samples. In MOLZ extracts, a storage duration-dependent decrease in the relative abundance was observed for Bacteroidetes whose average proportion dropped to 0.08% at month 18. As a consequence, the Firmicutes to Bacteroidetes 16S rRNA gene ratio continuously increased during storage at −80°C, while, with the exception of two measurements, it remained rather stable in NS-MAG samples (Figure 3C).

When considering “absolute abundances,” comparison of MOLZ to NS-MAG samples revealed that Firmicutes was only slightly decreased at all time points, excepted at 18 months of frozen storage. Specific decrease of Firmicutes in MOLZ samples at 18 months was due to late fragilization of several OTUs (marked with an asterisk on Figure 2B). Absolute abundances of all other phyla were far lower in MOLZ than in NS-MAG samples (Figure 3B).

**Figure 4:**
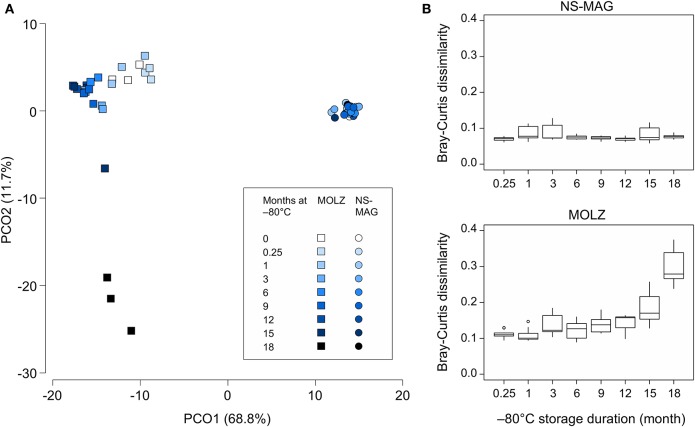
Overall microbiota changes during frozen storage. **(A)** PCoA of Bray-Curtis similarity matrix constructed using the square-root-transformed relative abundance of OTUs. **(B)** Bray-Curtis dissimilarity between fresh and frozen samples. Each boxplot summarizes nine pairwise comparisons between the three fresh aliquots of fecal suspension and the three aliquots processed following indicated frozen storage period.

To assess differences in overall taxonomic profiles of NS-MAG and MOLZ extracts, and to investigate the effect of frozen storage duration, we constructed the Bray-Curtis similarity matrix based on square-root-transformed OTU relative abundance. PCoA of Bray-Curtis similarities revealed sample clustering according to DNA extraction method (Figure 4A). In the PCoA plot, NS-MAG samples clustered more tightly as compared to MOLZ samples, which were more scattered, especially at 18 months of frozen storage. Consistent with this, Bray-Curtis dissimilarity between baseline (fresh) and later (frozen) samples increased only slightly up to month 12 in MOLZ samples, and then rose markedly from month 15. In contrast, Bray-Curtis dissimilarity remained rather stable in NS-MAG samples throughout the storage period (Figure 4B).

## Discussion

Our study aimed at detecting possible long-term frozen-storage effect on stool sample microbial composition through NGS analysis. The two DNA extraction methods used to sense these changes, resulted in different taxonomic profiles.

The primary purpose of using MOLZ DNA extraction procedure is the removal of host DNA and enrichment of intact microorganisms. This procedure is generally not intended for the extraction of microbial DNA from samples with high microbial mass such as fecal samples. We used this protocol (including a proteinase K pretreatment) to get an estimate of cells able to resist harsh conditions that fecal transplants may face during a freezing period and then, once released in the host intestine by FMT; these include the presence of host antimicrobial proteins and peptides, bacteriocins and antibacterial metabolites of bacterial origin (Ostaff et al., 2013; Kanjan and Hongpattarakere, 2016; Dicks et al., 2018). To the best of our knowledge, no attempts were made to impose stress conditions to fecal transplants and therefore distinguished between live and dead/injured bacteria.

Differences in relative and absolute abundance of bacterial phyla were observed between NS-MAG- and MOLZ-treated samples, both for fresh and frozen conditions. In the MOLZ procedure, according to the manufacturer guidelines, the proteinase K treatment is adapted to solubilize solid human tissues and release bacterial biofilm, whereas the CM buffer, containing a chaotropic agent, selectively disrupts human cells. Inclusion of a DNase treatment in the MOLZ protocol removes DNA of targeted human cells but also cell-free DNA and DNA from dead, damaged and most fragile bacteria. Indeed, the susceptibility to proteinase K cell lysis depends on the cell wall composition and structure (Moore et al., 2004). Similarly, the CM buffer (the composition of which is not fully disclosed for proprietary reasons) contains substantial proportion of a chaotropic agent (guanidine hydrochloride) that has greater efficacy in lysing Gram-negative bacteria and mammalian cells than Gram-positive bacteria (Boom et al., 1990).

The lower levels of Bacteroidetes in MOLZ samples as compared to NS-MAG samples may be attributed to removal of extracellular DNA and DNA from cells susceptible to lysis by MOLZ protocol. Further decrease in Bacteroidetes abundance during storage at −80°C reflects damaging effects occurring during cryopreservation.

In contrast to Bacteroidetes and other phyla, Firmicutes showed good resistance to MOLZ treatment and a weak loss up to 12 months of cryopreservation. OTUs associated to known butyrate-producing bacteria (Reichardt et al., 2014), considered as health promoters (Hamer et al., 2008; Louis and Flint, 2009), showed relatively little changes in relative abundance when compared to the fresh samples. These OTUs belonged to *Eubacterium hallii, Anaerostipes hadrus, Coprococcus catus, Coprococcus comes, Roseburia inulivorans*, and *Faecalibacterium*. Some of these bacteria also produce propionate, another short-chain fatty acid with health-promoting effects (Reichardt et al., 2014). Propionate producers *Eubacterium siraeum, Ruminococcus bromii*, and *Blautia obeum*, also showed little drop in abundance during 12 months of cryopreservation. Among most abundant butyrate and/or propionate producing bacteria identified in our study, only *Phascolarctobacterium faecium, which is a* propionate producer (Ogata et al., 2019), showed >50% reduction (56%) in the relative abundance in the first 12 months of cryopreservation. Our results suggest that members of the phylum Firmicutes better support stress conditions and thus would have better chances of survival in the FMT recipients.

A previous study reported that following 6 months of frozen storage of stools in 10% glycerol, CFU counts of six bacterial groups (bifidobacteria, *Escherichia coli*, total coliforms, lactobacilli, total anaerobic bacteria, or total aerobes) were similar to baseline values (Costello et al., 2015). Similarly, Bircher et al. (2018) showed that the gut commensal *Bacteroides thetaiotaomicron* (phylum Bacteroidetes) was resistant to cryopreservation for 3 months in terms of CFU counts and membrane integrity. Another study, based on a PMA treatment combined with qPCR, showed a reduced overall viability but no significant changes in composition of viable microbiota frozen fecal samples (Papanicolas et al., 2019). In these studies, bacterial or fecal suspensions were prepared under anaerobic conditions and were not exposed to the chemical or enzymatic stress similar to those used in the MOLZ protocol.

Recently, Takahashi et al. (2019) showed that in a murine model, enteric colonization by Bacteroidetes, but not that of Firmicutes, was reduced by 1-month frozen storage of a fecal suspension, which is consistent with a higher sensitivity of Bacteroidetes to stressful conditions, as suggested in the present study. Interestingly, in the study of Takahashi et al., *in vitro* bacterial viability, assessed by propidium monoazide (PMA) 16S community profiling, was not significantly changed during 1-month cryopreservation.

With no stress, as in the NS-MAG extraction method, which allow extraction of both free and intracellular DNA, the samples analysis exhibited a remarkable taxonomic stability, in line with a recent report showing little differences in microbial communities after a 5-year frozen storage at −80°C (Tap et al., 2019).

In conclusion, MOLZ protocol is sensitive enough to detect changes that occur during frozen storage, because it includes pre-treatments steps which could favor the lysis of the most fragile bacteria. In contrast, the NS-MAG protocol, which takes into account free DNA and DNA from dead, damaged and undamaged microbes, was found to be insensitive to change that can occur during frozen storage. NS-MAG and MOLZ appear to be complementary. Performing the two techniques makes it possible to highlight the complete taxonomic profile (NS-MAG) and possibly, to evaluate the level of cell preservation under prolonged frozen storage (MOLZ).

## Data Availability Statement

The datasets generated for this study can be found in the ENA, study number PRJEB33794.

## Ethics Statement

The studies involving human participants were reviewed and approved by Swissmedic. The patients/participants provided their written informed consent to participate in this study.

## Author Contributions

JS, SH, BH, and VL designed the study. SD, NG, MG, and SL performed the experiments. SD, YC, and VL drafted the manuscript. JS, SH, and BH edited the manuscript.

### Conflict of Interest

The authors declare that the research was conducted in the absence of any commercial or financial relationships that could be construed as a potential conflict of interest.
